# SARS-CoV-2 tropism, entry, replication, and propagation: Considerations for drug discovery and development

**DOI:** 10.1371/journal.ppat.1009225

**Published:** 2021-02-17

**Authors:** Nicholas Murgolo, Alex G. Therien, Bonnie Howell, Daniel Klein, Kenneth Koeplinger, Linda A. Lieberman, Gregory C. Adam, Jessica Flynn, Philip McKenna, Gokul Swaminathan, Daria J. Hazuda, David B. Olsen

**Affiliations:** 1 Department of Genetics and Pharmacogenomics, Merck & Co., Inc., Kenilworth, New Jersey, United States of America; 2 Exploratory Science Center, Merck & Co., Inc., Cambridge, Massachusetts, United States of America; 3 Department of Infectious Diseases and Vaccines, Merck & Co., Inc., West Point, Pennsylvania, United States of America; 4 Department of Computational and Structural Chemistry, Merck & Co., Inc., West Point, Pennsylvania, United States of America; 5 Department of Pharmacokinetics, Merck & Co., Inc., West Point, Pennsylvania, United States of America; 6 Department of Quantitative Biosciences, Merck & Co., Inc., West Point, Pennsylvania, United States of America; 7 Discovery Biology & Translational Medicine, Merck & Co., Inc., West Point, Pennsylvania, United States of America; University of Alberta, CANADA

## Abstract

Since the initial report of the novel Coronavirus Disease 2019 (COVID-19) emanating from Wuhan, China, Severe Acute Respiratory Syndrome Coronavirus 2 (SARS-CoV-2) has spread globally. While the effects of SARS-CoV-2 infection are not completely understood, there appears to be a wide spectrum of disease ranging from mild symptoms to severe respiratory distress, hospitalization, and mortality. There are no Food and Drug Administration (FDA)-approved treatments for COVID-19 aside from remdesivir; early efforts to identify efficacious therapeutics for COVID-19 have mainly focused on drug repurposing screens to identify compounds with antiviral activity against SARS-CoV-2 in cellular infection systems. These screens have yielded intriguing hits, but the use of nonhuman immortalized cell lines derived from non-pulmonary or gastrointestinal origins poses any number of questions in predicting the physiological and pathological relevance of these potential interventions. While our knowledge of this novel virus continues to evolve, our current understanding of the key molecular and cellular interactions involved in SARS-CoV-2 infection is discussed in order to provide a framework for developing the most appropriate in vitro toolbox to support current and future drug discovery efforts.

## Introduction

Coronaviruses, named for their crown-like spiked surface, are genetically diverse and can infect multiple animal species, including bats, pigs, cats, rodents, and humans [1]. Coronaviruses are divided into 4 genera: alpha, beta, gamma, and delta. Only alpha and beta coronaviruses are known to infect humans, resulting in pathology ranging from upper respiratory symptoms typical of the common cold to life-threatening lower respiratory disease. The common cold-causing coronaviruses 229E and OC43 were first discovered in the mid-1960s, with 2 additional coronaviruses, NL63 and HKU1, identified in 2004 and 2005, respectively. All are ubiquitous human pathogens [2]. From 2003 to mid-2019, 2 beta coronaviruses of zoonotic origin have caused outbreaks of severe respiratory disease: Severe Acute Respiratory Syndrome Coronavirus (SARS-CoV) and Middle East Respiratory Syndrome Coronavirus (MERS-CoV). SARS-CoV emerged in Asia in February 2003 and spread to 26 countries before the outbreak was contained [3,4]. Over 8,000 people were infected with a case fatality rate of approximately 10% [5]. MERS-CoV first appeared in 2012 with early cases emanating from Saudi Arabia and Jordan. Infections are still occurring and have been reported in 27 countries, with the majority of cases isolated to the Arabian Peninsula [6]. While human-to-human transmission for MERS-CoV is rare, the case fatality rate is greater than 30% [3,7].

In December 2019, an outbreak of fever and respiratory illness of unknown cause was reported in Wuhan, China [8], and by mid-January 2020, the etiologic agent had been identified as another newly emergent beta coronavirus, Severe Acute Respiratory Syndrome Coronavirus 2 (SARS-CoV-2) [9,10]. While many infected with SARS-CoV-2 are asymptomatic or develop mild disease, for others, COVID-19 may have potential long-term sequelae; and in vulnerable populations like the elderly and those with underlying medical conditions, it may cause significant morbidity and result in severe respiratory distress, hospitalization, and even death [11]. Since that time, SARS-CoV-2 has spread globally, prompting the World Health Organization (WHO) to declare the novel coronavirus disease, Coronavirus Disease 2019 or COVID-19, a pandemic in March 2020. In just 12 months, the virus has resulted in a major global health crisis with over 81 million COVID-19 cases across 190 countries, over 1,777,000 deaths, and an estimated case fatality rate of approximately 2.6% [12].

Aside from the intravenously administered antiviral drug remdesivir in patients with severe COVID-19 illness, there are no therapeutic agents approved for treatment of SARS-CoV-2 infection or disease [13]. A multicenter evaluation of 4 repurposed antiviral drugs (remdesivir, hydroxychloroquine, lopinavir, and interferon β 1a) reported by WHO noted no effect on overall mortality initiation of ventilation and duration of hospital stay [14]. A recent subgroup analyses suggested that early glucocorticoid use in patients with markedly elevated C-reactive protein levels (≥20 mg/dL) was associated with a significant reduction in mortality or mechanical ventilation, whereas glucocorticoid treatment in patients with lower C-reactive protein levels was associated with worse outcomes [15]. As the SARS-CoV-2 pandemic continues, there is an urgent need to develop effective therapeutics to limit further spread. Early attempts to identify efficacious therapeutics for COVID-19 have mainly focused on drug repurposing efforts wherein existing clinically advanced or marketed drugs are screened for antiviral activity against SARS-CoV-2 in vitro in cellular infection systems. While such screens have yielded intriguing hits, questions have arisen around the physiological and pathological relevance of infecting immortalized cell lines derived from non-pulmonary or gastrointestinal origins. Specific questions have arisen around the mechanisms of viral attachment and entry into human cells which may vary in cells from different tissue origins. In addition, screening cell lines may have limited intracellular machinery, such as catabolizing enzymes, which are a key component of the primary cell of infection in human patients. It is therefore of paramount importance to enhance our understanding of the key molecular and cellular interactions involved in SARS-CoV-2 infection in order to develop appropriate in vitro tools to support current and future drug discovery efforts.

### Scope/prior reviews

The purpose of this article is to review key aspects of SARS-CoV-2 biology, including determinants of virus entry into multiple cell lines and systems permissive for virus growth, tissue tropism, and host genes affecting viral entry, propagation, and nucleotide prodrug import and conversion for SARS-CoV-2, with the purpose of facilitating and enabling drug discovery efforts.

For a more comprehensive review of entry mechanisms and proteases processing in coronaviruses, the reader is directed to other recent articles [16–19]. Extensive reviews are available on MERS-CoV [20], SARS-CoV [21,22], and SARS-CoV-2[19] inhibitors. We discuss SARS-CoV-2 repurposing screens in the “Entry mechanisms and proteases” section.

### Entry mechanisms and proteases

To date, initial screening to identify SARS-CoV-2 antivirals has largely utilized the Vero E6 African green monkey kidney cell line as the host cell for cytopathic effect (CPE) inhibition assays. In addition to being deficient in expression of angiotensin converting enzyme 2 (ACE2) and TMPRSS2, intrinsic nonspecific endocytic viral uptake mechanisms are responsible for viral entry in Vero E6 and a variety of other cell types [23–26]. Thus, a wide variety of molecules that modulate endosomal–lysosomal maturation and autophagy pathways have been found to exhibit potent antiviral activity in Vero E6 cells that may not translate to primary lung epithelial cells. This raises the possibility that the antiviral inhibition exhibited by many of these compounds against SARS-CoV-2 in Vero E6 screens could be an in vitro activity specific to highly endocytic cells and may not be relevant nor translatable as SARS-CoV-2 therapeutics. As further elaborated below, the SARS-CoV-2 virus has been demonstrated to deliver viral RNA directly across the plasma membrane without significant involvement of endocytic uptake.

Infection of mammalian lung epithelial cells by SARS-CoV-2, as well as by other coronaviruses, starts with binding of the virus through its spike protein to a specific receptor on the cell surface. The cellular receptor for SARS-CoV-2, and for the related virus SARS-CoV, is human ACE2 [16,27,28], which is expressed on epithelial cells of the lung and intestine, and to a lesser extent, in the kidney, heart, adipose, and both male and female reproductive tissues [29–35]. Binding to the receptor is followed by activation of the spike protein through proteolytic cleavage by a host protease near the junction between its S1 and S2 domains [17,36]. Insertion of the newly liberated S2 domain N-terminus into the cell membrane leads to fusion of the viral and cellular membranes, resulting in transfer of the viral RNA into the host cell cytoplasm where viral replication can occur [17,36].

**Fig 1:**
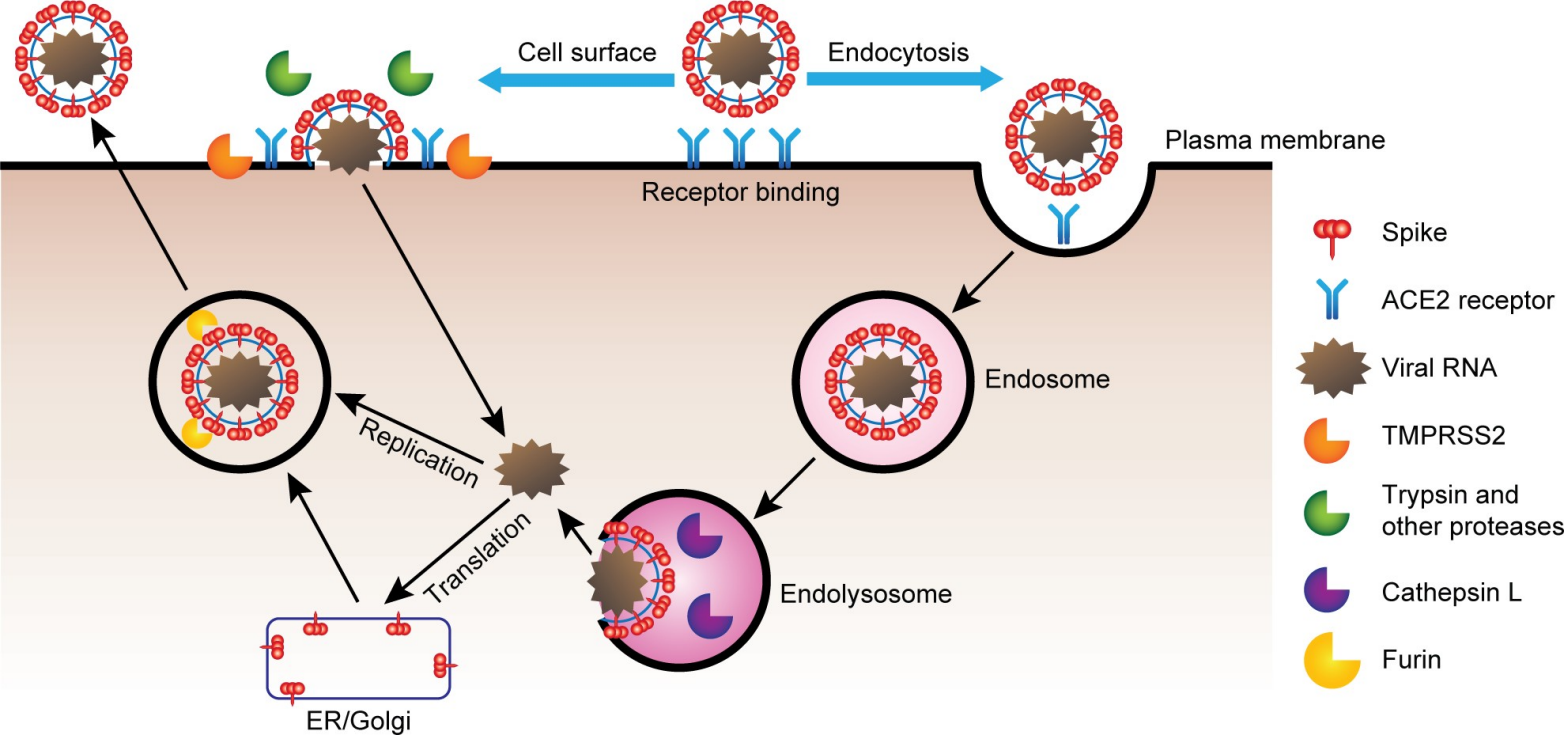
SARS-CoV-2 entry mechanisms. Viral coat spike protein binds to ACE2, and in some cases, perhaps NRP1, on responsive cells. Virus spike protein is either processed by TMPRSS2 and other serine proteases facilitating cell surface entry or endocytosed into endosomes where spike is processed by CTSL in the lysosome. Viral RNA released from TMPRSS2-mediated entry or endosome release is replicated as partial and complete genome copies and translated in the ER to form new SARS-CoV-2 virions. Processing of spike protein by furin occurs prior to release of new viruses into the extracellular environment. ACE2, angiotensin converting enzyme 2; CTSL, cathepsin L; ER, endoplasmic reticulum; NRP1, Neuropilin 1; SARS-CoV-2, Severe Acute Respiratory Syndrome Coronavirus 2.

While the steps outlined above are considered essential for infection of host cells by coronaviruses, this description omits key aspects of the viral entry process, namely the nature of the host protease(s) responsible for activating (or priming) the spike protein for fusion and the cellular location of the fusion event itself. Work on multiple coronaviruses over the last 20 years, and largely confirmed for SARS-CoV-2 within the last several months, shows that coronaviruses enter host cells through 1 of 2 distinct pathways: (i) the cell surface pathway following activation by serine proteases such as TMPRSS2; or (ii) the endocytic pathway within the endosomal–lysosomal compartments including processing by lysosomal cathepsins (Fig 1) [16–18]. A new study argues that coronaviruses use a lysosomal exocytosis pathway for release [37]. The contribution of each pathway in a given cell type depends largely on the expression of proteases, in particular TMPRSS2. When TMPRSS2 (or other serine proteases such as TMPRSS4 or human airway trypsin-like protease [HAT]) is expressed, the early entry pathway is preferred, whereas in the absence of this protease, the virus relies on the late pathway involving endocytosis and activation by cathepsin L (CTSL) [27,38].

An understanding of which viral entry pathway is prevalent in specific cell types is paramount not only to understanding coronavirus biology, but also to the proper interpretation of cell-based genetic and small-molecule screens. To date, screens aimed at identifying marketed or clinically advanced drugs for potential repurposing for the treatment of COVID-19 have relied on the use of immortalized kidney epithelial cell lines such as Vero E6 (African green monkey) and 293T (embryonic human) [39–42]. Such screens have yielded multiple hits known to block or impair endocytosis or endosomal maturation [39,40,42,43]. This is not surprising since Vero E6 and 293T cells do not express the cell surface protease TMPRSS2; therefore, coronavirus infection in these cells is dependent on the late endocytic/cathepsin-mediated entry pathway. Thus, agents that interfere with this pathway, including cathepsin inhibitors, agents that prevent endosomal acidification (upon which the cathepsins are dependent for activity), and inhibitors of endosomal maturation (e.g., the PIKfyve inhibitor apilimod) can block viral entry in these cells [38,44,45]. Importantly, heterologous expression of TMPRSS2 in Vero E6 and HeLa cells abrogates the pharmacological efficacy of cathepsin inhibitors by enabling the cell surface viral entry pathway [38,46]. Similarly, pre-activation of virus with the serine protease trypsin also enables the cell surface entry pathway, even in cells that do not express TMPRSS2 [45,47,48]. Consistent with these observations, cells that express TMPRSS2 endogenously, including cells derived from the lung and intestinal epithelium, are also susceptible to coronavirus entry via the cell surface pathway. In these cells, cathepsin inhibitors are only partially effective, TMPRSS2 inhibitors such as camostat and nafamostat are significantly more effective, and a combination of the 2 types of protease inhibitors is maximally effective in preventing coronavirus infection [27,38,49–52]. These data suggest that coronaviruses can enter these cells via both pathways, with a preference for the cell surface pathway.

These cellular data notwithstanding, it is still a matter of debate whether individual inhibition of the cell surface or endocytic pathways will provide therapeutic benefit against COVID-19. SARS-CoV-2 infects a broad variety of cell types [46,53,54], although it seems reasonable to surmise that epithelial cells of the respiratory and gastrointestinal tracts are among the first to encounter the virus in a clinical setting. In vivo studies with the related coronavirus SARS-CoV supports the notion that inhibitors of the cell surface entry pathway may be at least partially protective. Genetic [55] and pharmacological [56] blockade of TMPRSS2 provided partial protection against SARS-CoV in mouse models of infection. In the latter study, the broad cathepsin inhibitor SMDC256160 provided no benefit, either alone or in combination with camostat. Further studies will be required, particularly in SARS-CoV-2 infection models, to further define the contribution of each viral entry pathway to COVID-19 pathology. Notably, it has recently been proposed that SARS-CoV-2 entry blockade may require inhibition of both the cell surface and endocytic pathways [57].

### TMPRSS2 and furin in cell surface entry

The TMPRSS2 gene encodes a type II transmembrane serine protease (TTSP) that was originally discovered more than 2 decades ago [58] and subsequently shown to be regulated by androgen and highly expressed in prostate epithelium [59]. TMPRSS2 is localized to the plasma membrane via a single-pass transmembrane helix near its N-terminus. The enzyme can also undergo autocleavage at Arg255 yielding a 32-kDa secreted form of the protease [60]. Mice lacking the TMPRSS2-encoded protease (TMPRSS2[-/-]) show no discernable abnormal phenotype, suggesting that the protease does not serve an essential nonredundant function [61], an observation which enabled later investigation of TMPRSS2’s role in mouse models of coronavirus disease.

Our current understanding of the relationship between TMPRSS2 and SARS-CoV-2 infection has seen rapid progress in the first few months since the emergence of SARS-CoV-2. Much of this advancement was built on more than a decade of studies beginning from the first evidence that TMPRSS2 expression correlates with SARS-CoV infection in lung tissue and leads to activation of the spike protein, enabling membrane fusion [62]. Studies using cell fusion assays indicated that TMPRSS2 expression on target cells, rather than virus producing cells, is critical for spike protein activation, supportive of spatial and temporal constraints on TMPRSS2 action at the plasma membrane during the early steps of cell surface viral entry. Two sites of cleavage on SARS-CoV spike—R667, located at the S1/S2 cleavage site, and R797, located at the S2’ cleavage site—have been suggested to be relevant sites of action of TMPRSS2, as well as other serine proteases that can prime the spike protein in cell culture, such as trypsin and HAT. Mutation of R797 at S2’ abrogates TMPRSS2-dependent activation of the spike protein, and this site is highly conserved across coronaviruses, suggesting that is functionally relevant to TMPRSS2-dependent cell surface entry in SARS-CoV-2.

The pro-protein convertase furin has long been known to play a role in viral entry, and recent data support a role of this enzyme, specifically in TMPRSS2-mediated cell surface entry. Processing of the spike protein by furin at the S1/S2 cleavage site is thought to occur following viral replication in the endoplasmic reticulum Golgi intermediate compartment (ERGIC) [63]. Underscoring the complex role of furin in viral fusion and entry, the S1/S2 furin cleavage site is present in SARS-CoV-2 and MERS but not in SARS-CoV [64]. Furthermore, the SARS-CoV-2 furin S1/S2 site is rapidly lost upon passaging in Vero E6 cells [65], and a milder SARS-CoV-2 strain isolate ZJ01 was demonstrated to have lost this site as well [30]. Furin can also cleave the spike protein at the fusion activating the S2’ site during biogenesis in MERS-CoV and SARS-CoV [30,36,65–67]. Overall, current available data support a plausible model for SARS-CoV-2 spike processing wherein furin-mediated cleavage at the S1/S2 site pre-primes the spike protein during biogenesis, facilitating subsequent activation for membrane fusion by a second cleavage event at S2’ by TMPRSS2 following ACE2 receptor binding on target cells [46,50].

The VEGF-A receptor Neuropilin 1 (NRP1) was quite recently shown to be a host factor receptor for furin-cleaved SARS-CoV-2 spike peptide [68–71]. Blockade of NRP1 reduces infectivity and entry, and alteration of the furin site leads to loss of NRP1 dependence. Deletion of the furin peptide in spike leads to reduced replication in Calu3, augmented replication and improved fitness in Vero E6, and attenuated disease in a hamster pathogenesis disease model [70].

More recently, in vivo evidence for the relevance of TMPRSS2 in mouse models for SARS-CoV and MERS-CoV infection was provided from TMPRSS2(-/-) mice showing reduced disease severity [55]. TMPRSS2 shRNA knockdown studies provide evidence for a specific and nonredundant role in SARS-CoV-2 infection [72].

### Lysosomal cathepsins and endocytosis

While the evidence outlined above makes clear the role of TMPRSS2 and other serine proteases in activating the coronavirus spike protein for plasma membrane fusion, in vitro studies using various cell culture systems have demonstrated an alternative endosomal–lysosomal pathway for viral entry. Early studies demonstrated the sensitivity of SARS-CoV replication in cell culture to lysosomotropic agents, followed by additional studies dissecting the role of cathepsins for processing and activating the spike for membrane fusion. The availability of highly potent and specific small-molecule cathepsin inhibitors was key to dissecting the molecular events involved in this pathway and ascribing the relevant functional effect to CTSL, 1 of 11 cathepsins in humans [73,74]. Additional studies localized the site of CTSL cleavage on SARS-CoV spike protein to T678, 11 amino acids carboxyl terminus to R667 of the furin cleavage site. The observation that the CTSL cleavage site is located 120 amino acids upstream of the S2’ site cleaved by TMPRSS2 exposes a gap in our understanding of the molecular events involved in the endosomal entry pathway. Previous studies suggested the possibility of another protease that may cleave at S2’ in the low pH environment of endosomes/lysosomes to fully activate the membrane fusion potential of the spike [36], or alternatively, differences in lipid composition between plasma membranes and endosomal/lysosomal membranes may render the latter more amenable to viral fusion [75]. Despite this discrepancy in our current understanding of endocytic viral entry, it is clear that coronaviruses in general, and SARS-CoV-2 in particular, are capable of establishing robust infection through endosomal entry in commonly used in vitro cell culture systems.

### Cell line tropism/expression

With the complexity of multiple entry mechanisms of SARS-CoV-2, selection of optimal cell line(s) for compound evaluation and screening is imperative, especially in studies looking for broad antiviral mechanisms of action beyond inhibition of the viral nonstructural proteins. Toward this end, studies to better understand the cellular tropism of the virus have been reported. A Vesicular Stomatitis Virus (VSV) pseudotyped virus bearing SARS-CoV-2 spike protein or SARS-CoV spike protein and a panel of well-characterized human and animal cell lines was used to determine the broader tropism of the virus [46]. The spectrum of cell lines susceptible to virus infection was similar for SARS-CoV and SARS-CoV-2 spike protein with entry supported in A549, BEAS-2B, Calu-3, H1299, 293T, Huh7, Caco-2, Vero E6, and MDCK2 cell types [27]. Additional efforts to understand viral tropism included monitoring the infectivity of virus for 120 hours with 0.1 multiplicity of infection (MOI) of SARS-CoV-2 HKU-001a across a panel of 9 human and 11 nonhuman cell lines [53]. Similar to the results observed with a pseudotyped virus, the most robust infection among the human cell lines was observed for Calu-3 (pulmonary) and Caco-2 (intestinal) cells with infection also observed in Huh7 (hepatic), 293T (renal), and to a lesser extent, U251 (neuronal) cells. Among the 11 cell types found permissive to infection in the study, CPE was observed 120 hours postinfection only in Vero E6 and FRhK4 (rhesus kidney), with damage including cell rounding, detachment, degeneration, and syncytium formation. Even up to 7 days postinfection, CPE was not observed in Calu-3, Caco 2, LLCMK2, PK-15, and RK-13 cells with SARS-CoV-2.

At a higher MOI of one, a study of proteomics kinetic changes on infection showed both viral replication and CPE in Caco-2 cells [76]. By monitoring protein expression levels, the authors of this study observed that infection results in the reshaping of central cellular pathways, such as translation, splicing, carbon metabolism, and nucleic acid metabolism. This assay was modified by lowering the MOI from 1 to 0.01 and increasing incubation time from 24 to 48 hours to allow for several cycles of viral replication to screen for antivirals in a high-content imaging assay [77]. From a screen of 5,632 compounds including greater than 3,400 clinical candidates, the authors highlighted 6 compounds which had previously been reported to be active against SARS-CoV-2, SARS-CoV, or MERS in antiviral assays in Vero E6, Calu-3, or BHK21 (hamster kidney fibroblast) cells, with the pattern of inhibition matching more closely to results in human Calu-3 cells than in nonhuman Vero E6 and BHK21 cells. For example, camostat and nafamostat, inhibitors of TMPRSS2, which plays a role in the cell surface pathway for viral cellular entry, were measured to be 100x more potent against SARS-CoV-2 in Caco-2 cells than in Vero E6 cells. However, compounds like remdesivir, lopinavir, and mefloquine were relatively consistent across cell types.

Further delineating the different mechanisms involved in SARS-CoV-2 entry and infection, Dittmar and colleagues applied a high-content imaging assay to compare the antiviral activity of a panel of approximately 3,000 compounds [39]. Less than 1% infection was observed in several cell types including A549, Calu-1, Huh7, HepG2, HaCaT, IMR90, NCI-H292, CFBE41o, and U2OS cells; however, robust infection was observed in the human hepatocyte cell line Huh7.5, lung-derived Calu-3 cells, and Vero CCL81 cells. Among the 3 cell types, compounds with different mechanisms of action were observed to have variable antiviral activity. For example, inhibitors of endosomal entry, such as the cathepsin inhibitor Z-FA-FMK and PIKfyve inhibitor apilimod, were active in Huh7.5 and Vero E6 cells but not Calu-3 cells. In contrast, camostat, an inhibitor of the plasma membrane protease TMPRSS2, was active in Calu-3 cells but not Huh7.5 and Vero E6 cells, further highlighting the importance of understanding the translatability of a cellular model of infection.

Viral tropism of SARS-CoV-2 for various cell types likely reflects differential expression of key host proteins involved in viral attachment and entry. Table 1 summarizes publicly available expression data for the SARS-CoV-2 cellular receptor ACE2 and for 5 proteases involved in viral fusion and entry (furin, TMPRSS2, CTSL, elastase, and trypsin). While CTSL is expressed in all lines examined, ACE2 and TMPRSS2 levels vary, explaining the use of heterologous expression of these genes in prior studies involving Vero E6 and other cell lines.

**Table 1:** Cell line expression level of ACE2 receptor and 5 proteases.

Cell line	Species	Tissue	NCBI GEO GSM	ACE2	Furin	TMPRSS2	CTSL	Elastase	Trypsin	
Vero E6	AGM	Kidney	GSM758842							
T84	Human	Colon	GSM24844							
Calu3	Human	Lung epithelial adenocarcinoma	GSM98974							
Caco2	Human	Colorectal adenocarcinoma	GSM24832							
Huh7	Human	Liver carcinoma	GSM523816							
293T	Human	Kidney embryonic	GSM871735							
U251	Human	Glioblastoma	GSM803750							
FRhK4	AGM	Kidney	GSM871739							
LLCMK2	AGM	Kidney	GSM871737							
BEAS-2B	Human	Lung bronchial epithelial	GSM157034							
H1299	Human	Lung NSCLC metastasis	GSM98990							
A549	Human	Lung epithelial adenocarcinoma	GSM2359842							
Calu1	Human	Lung bronchial epithelial	GSM1374431							
HepG2	Human	Liver carcinoma	GSM720616							
MRC5	Human	Lung fibroblast	GSM654536							
CRFK	Cat	Kidney	GSM3553405							
16HBE	Human	Bronchial epithelial	GSM417467							
HUVEC	Human	Vein epithelial	GSM385333							
		Expression	High	12.00	9.60	7.20	4.80	2.40	0.00	Low

Values are normalized log 2 expression with data obtained from NCBI Gene Expression Omnibus database (http://www.ncbi.nlm.nih.gov/geo/) [93].

ACE2, angiotensin converting enzyme 2; AGM, African green monkey; CTSL, cathepsin L; NSCLC, non-small cell lung cancer.

### Nucleotide/side import and conversion

Screening cell lines for evaluation of nucleotide prodrugs and nucleosides as inhibitors of viral RNA-dependent RNA polymerase (RdRp) must consider their ability to import and convert compounds to active nucleoside triphosphate (NTP) metabolites [78]. Remdesivir [79–81] is a phosphoramidate protide drug [82] requiring import and conversion in host cells to inhibit the replication essential SARS-CoV-2 RdRp.

Cellular uptake of modified nucleosides largely depends on expression of concentrative membrane transporters (cNTs) or equilibrative/facilitative membrane transporters (ENTs) [83,84]. Intracellular conversion to nucleosides to nucleotides is mediated by cellular kinase phosphorylation, and deficient expression can reduce antiviral potency [39,85,86].

Some prodrugs require expression and enzymatic conversion by esterases and lysosomal cathepsin A.

**Fig 2:**
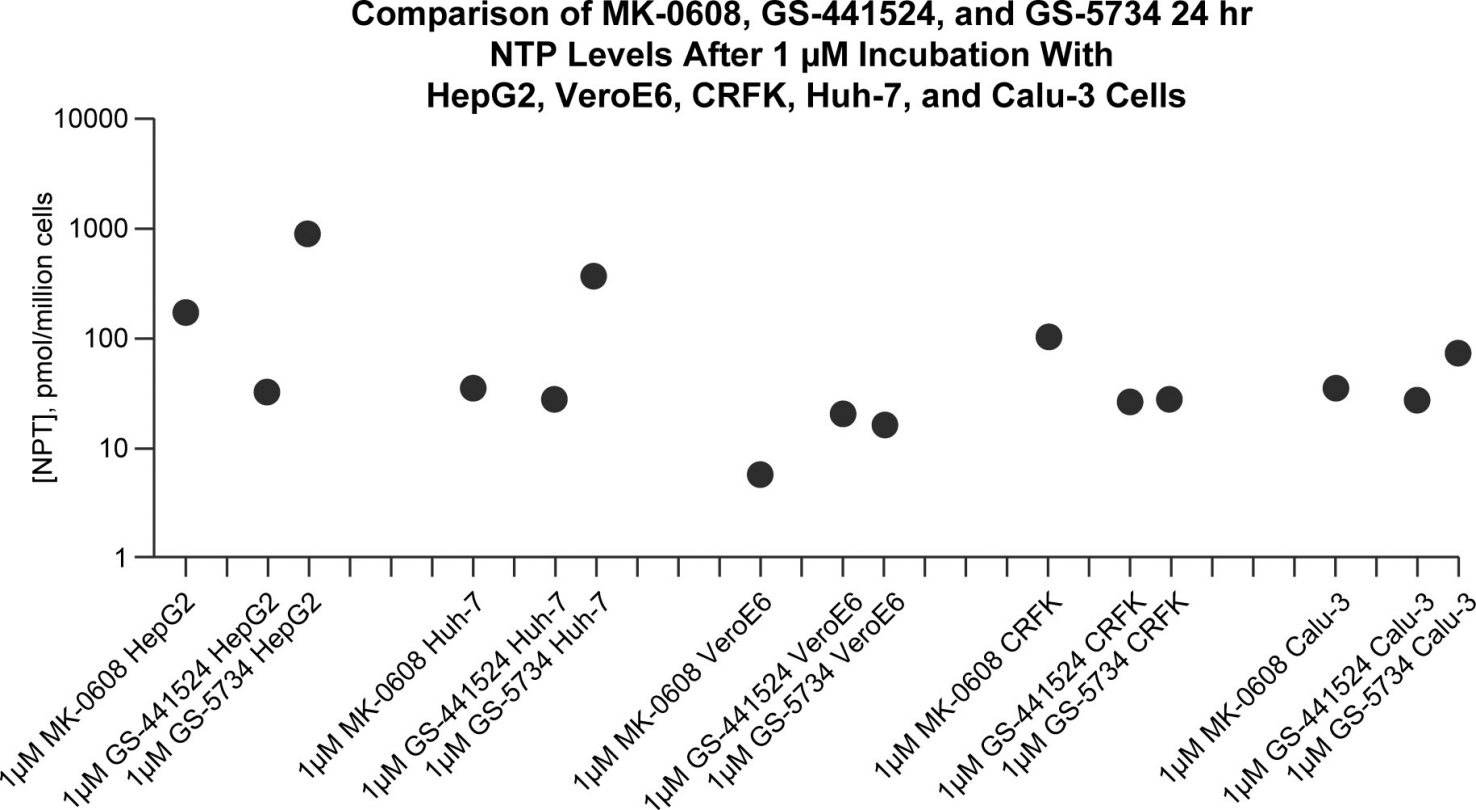
Nucleotide phosphorylation. The 24-hour intracellular NTP concentration of adenosine antiviral drug leads (MK-0608, Gilead Sciences remdesivir prodrug GS-5734, and Gilead Sciences remdesivir parent nucleoside GS-441524) after incubation at 1 μM illustrates the apparent deficiency of Vero E6 cells with regard to conversion to the active triphosphate form as compared to other cell lines studied. NTP, nucleoside triphosphate.

We therefore tested 5 cell lines for ability to phosphorylate adenosine ribonucleosides MK-0608 and GS-441524 (parent nucleoside of remdesivir) compared with remdesivir prodrug GS-5734 and demonstrated that host cells have differential capability for drug activation (Fig 2). Hepatocyte cell lines HepG2 and Huh7 exhibited significantly better activation of monophosphate phosphoramidite prodrug GS-5734 than African green monkey Vero E6 kidney cells, Crandell-Rees feline kidney (CRFK) cells, or human Calu-3 lung cells due to increased cathepsin A expression in hepatocytes compared with extrahepatic cells [87].

### Primary cells/model systems

Cell lines provide a rapid method to screen and gather data in a high-throughput manner, but there are limitations in their translatability. Primary cell culture systems may more accurately model relevant cellular physiology. Insight into the SARS-CoV-2 course of infection has been gleaned using single cell sequencing results from the Human Atlas (noninfected individuals) to identify transcriptional expression of viral receptors. Protein staining of these receptors is a better indicator of actual surface expression, but such data are currently sparse. Analysis of RNA from infected patient tissue samples provides a snapshot in time for which we often do not know the timing of the onset of infection. Primary in vitro model systems allow for the testing of hypotheses in human tissues and allow us to gather temporal information about gene/protein expression.

Many studies have reported high levels of ACE2 and TMPRSS2 co-expression in lung AT2 cells utilizing data from public databases and lung resections [29,88,89]. Lung air–liquid interface (ALI) cultures derived from various regions of the lung from healthy individuals showed the greatest co-expression of ACE2 and TMPRSS2 in transient secretory cells of the bronchial branches. It was also shown that there is triple expression of ACE2, TMPRSS2, and FURIN when looking across lung tissue, with a preference for co-expression of at least 2 of the receptors [29]. In a recent study, single cell (sc) RNA sequencing (seq) was used to follow viral RNA on a per cell basis over a time course of infected bronchial epithelial cells (ALI model) and found that the primary target of SARS-CoV-2 in the upper airway is ciliated lung cells, the cells responsible for removing mucus and foreign particles from the lung. As infection progresses, the virus infected basal and club cells over time, which they confirmed using electron microscopy. Additionally, an up-regulation was observed of type I and type III interferons (IFNs), IL-6, and the chemokines CXCL9, CXCL10, and CXCL11 (important for recruitment of T/NK cells) in infected cells and broad interferon-stimulated gene (ISG) up-regulation in infected cells as well as bystander cells [90]. While these single cell sequencing and single nucleus sequencing data provide much detail about the expression of RNA transcripts, an older study confirms co-staining of ACE2 and TMPRSS2 protein in lung sections by immunohistochemistry [91].

In contrast, others have reported that nasal epithelial cells have the highest expression of ACE2 in the respiratory tract. Additionally, by combing publicly available databases, it was found that the upper airway has the highest expression of ACE2-correlated genes, and genes with innate immune function are disproportionately represented [92]. Specifically, they reported that the nasal goblet cells have the highest expression of these immune genes.

While lung ALI cultures represent a more physiologically relevant primary model due to the exposure of the apical membrane to air, lung organoids have utility in studying SARS-CoV-2. Similar to lung ALI cultures, lung organoids are derived from basal stem cells of the lung (adult) or induced pluripotent stem cells (embryonic) and are grown in a 3D supportive matrix [93]. A benefit of using organoid cultures is that the stem cells can be propagated continuously and then terminally differentiated into more mature cell types of the epithelium allowing for more rapid expansion and differentiation of primary cells to numbers high enough to support higher throughput screening compared with ALI cultures, which take 21 to 28 days to reach maturity. One group reported using lung organoids to successfully screen the Food and Drug Administration (FDA)-approved Prestwick drug library with a luciferase-expressing SARS-CoV-2 pseudovirus [94]. Additionally, they confirmed 3 out of 4 hits in hPSC-derived colonic organoids.

While the mucosal surface is likely the entry site for SARS-CoV-2, examination of other tissues has revealed that high levels of ACE2/TMPRSS2 co-expression are present in the enterocytes of the ileum and colon, and the gut may have higher expression of these receptors than the lung [30,31]. The observation that some COVID-19 patients have gastrointestinal distress prior to developing respiratory symptoms as well as during disease progression may suggest a fecal–oral route of transmission [95,96]; evidence supporting this has been recently reviewed [97,98]. Similarly, gastrointestinal infection/distress was previously observed for the closely related SARS and MERS coronaviruses [99,100]. The transcriptional data are supported by endoscopic samples from a COVID-19 patient (esophagus, stomach, duodenum, and rectum) that displayed staining of the ACE2 receptor in the gastrointestinal epithelial cells, but not in the esophageal epithelium [101].

Studies have linked the observed gastrointestinal phenotype to the expression of ACE2+/TMPRSS2+ cells in the gut, with highest expression levels in the ileum, followed by the colon. More conclusively, infection of intestinal organoids with SARS-CoV-2 was successful, and they supported replication of the virus with enterocytes being the primary cell type targeted [31]. Interestingly, it was observed that type III IFN, but not type I IFN, was up-regulated following infection of colonoids with SARS-CoV-2, suggesting that this cytokine may play a protective role in this infection [102].

The intestinal organoid model has allowed further characterization of viral entry. Using a VSV-SARS-CoV-2 pseudovirus (expressing the SARS-CoV-2 spike protein), it was demonstrated that TMPRSS2 and TMPRSS4 are both necessary for fusogenic entry into duodenal enterocytes [103]. Additionally, the authors observed preferential infection of the apical membrane of the enterocytes, and imaging suggested polarized viral assembly and release from the apical membrane. These data demonstrate how data from primary cells/tissue can be further dissected when replicated in a physiologically relevant primary model system. The use of primary lung and gut model systems is proving to be very relevant for the study for SARS-CoV-2 as they more accurately replicate receptor expression and possibly the physiological responses seen in vivo.

It should be noted that other ex vivo systems, such as tissue explants, have been used to study viral respiratory infections and could likely be used to study SARS-CoV-2. Multispecies in vivo models are also rapidly being developed to better understand host responses to this virus. In vivo and ex vivo models that have been or are under development have recently been reviewed, including mouse, ferret, hamster, and nonhuman primate animal models [104,105].

### Innate immune cells

Immune dysfunction has been extensively characterized in COVID-19 patients, such as dysregulation of T cells, B-cells, and innate immune cells [106,107]. Specifically, increased prevalence and activation of innate immune cells has been observed in COVID-19 patients with severe disease [108]. Interestingly, it was reported that there are significant morphological and functional differences in monocytes derived from COVID-19 patients compared with healthy human donors [109]. Postmortem analyses of secondary lymphoid organs from COVID-19 patients have confirmed the expression of ACE2 receptor and the presence of SARS-CoV-2 nucleoprotein in CD169+ macrophages [110]. However, it is unclear if SARS-CoV-2 can infect and replicate in innate immune cells.

Varying degrees of viral entry and replication have been observed in primary innate immune cells infected with SARS-CoV-1, MERS-CoV, HCoV-OC43, and HCoV 229E [111]. Minimal expression of key receptors required for SARS-CoV-2 entry, including ACE2 and TMPRSS2 in innate immune cells such as monocytes and macrophages, has been recently reported in nonhuman primates[112] and humans [30]. In addition, monocytes can express high levels of CD147 [113], a potential receptor for SARS-CoV-2 entry [114].

Two recent publications have identified that CD14+ primary monocytes from healthy human donors are readily susceptible to SARS-CoV-2 infection. Pontelli and colleagues demonstrated this by infecting primary human monocytes ex vivo with SARS-CoV-2 and measuring viral antigens and dsRNA entities in infected cells [115]. They observed substantial reduction in viral entry when monocytes were infected in the presence of ammonium chloride, which suggested that the endocytic pathway is likely important. Similarly, Codo and colleagues observed infection of SARS-CoV-2 in primary human monocytes based on measurement of viral transcripts by qPCR [116]. Furthermore, they observed that SARS-CoV-2 infection of monocytes results in induction of glycolysis and increase in transcript levels of several genes including the main viral entry receptor ACE2.

Given the minimal expression of ACE2 receptor in monocytes, these observations are rather surprising. More in-depth virologic analyses of productive replication in monocytes will greatly bolster the observations in these 2 aforementioned publications. Furthermore, additional studies are needed to better understand if (i) monocytes/macrophages are exposed to SARS-CoV-2 via direct infection via ACE2 binding or through phagocytosis; (ii) ACE2 is induced in infected monocytes and bystander cells through secretion of inflammatory mediators; and (iii) expression of additional host entry receptors such as CD147 and Neuropilin-1 may be expressed in monocytes to facilitate infection in an ACE2-independent manner [69].

Continued evaluation of the susceptibility of innate immune cells to SARS-CoV-2 will greatly improve our current understanding of SARS-CoV-2-host interactions as well as the immunopathogenesis observed in COVID-19 patients.

### Concluding remarks

Phenotypic screening to identify novel inhibitors for infectious diseases is a costly and time-consuming endeavor. The most important decisions are the choice of cell line or model systems in which to conduct the screen and the availability of relevant secondary screens to eliminate irrelevant hits and prioritize those of potential interest. In this regard, assessing antiviral potency for SARS-CoV-2 viral replication should take into account the following key considerations. Expression of the ACE2 receptor and key host proteases will influence the nature of hits identified [117]. Screens in Vero E6 cells have identified agents affecting the endocytic pathway as having antiviral activity, but blockade of both endocytic and cell surface viral entry pathways may be required in the context of clinical infection. In addition to understanding the viral entry pathway(s) which SARS-CoV-2 uses in the specific cell types used for screening, consideration should be given to the degree of nucleotide prodrug activation and phosphorylation observed in the in vitro screening system, as clearly demonstrated recently [39]. Emerging model systems including ALI and organoid cultures are promising host mimetic systems for evaluating candidates, although these have their own limitations. Finally, the potential role of innate immune cells in infection is an additional important consideration. Selecting appropriate in vitro model systems reflecting multiple aspects of SARS-CoV-2 biology will be essential to optimize success in current and future drug discovery efforts.
